# Comparative Analysis of Chemical Composition of *Zanthoxylum myriacanthum* Branches and Leaves by GC-MS and UPLC-Q-Orbitrap HRMS, and Evaluation of Their Antioxidant Activities

**DOI:** 10.3390/molecules28155631

**Published:** 2023-07-25

**Authors:** Wei Dai, Liangqian Zhang, Liping Dai, Yuan Tian, Xinger Ye, Sina Wang, Jingtao Li, Qi Wang

**Affiliations:** 1 Experimental Center of Yunfu Campus, Guangdong Pharmaceutical University, Yunfu 527325, China; 2 Key Laboratory of Xinjiang Phytomedicine Resource and Utilization, Ministry of Education, School of Pharmacy, Shihezi University, Shihezi 832003, China; 3 College of Traditional Chinese Medicine Resources, Guangdong Pharmaceutical University, Yunfu 527325, China

**Keywords:** *Zanthoxylum myriacanthum* Wall. ex Hook. f., UPLC-Q-Orbitrap HRMS, GC-MS, chemical composition, antioxidant activities

## Abstract

*Zanthoxylum myriacanthum* Wall. ex Hook. f., a plant belonging to the Rutaceae family and the *Zanthoxylum* genus, is extensively utilized for its medicinal properties and as a culinary seasoning in China and Southeast Asian countries. However, the chemical composition and biological activities of *Z. myriacanthum* branches and leaves remain insufficiently explored. In this study, the volatile and non-volatile components of *Z. myriacanthum* branches and leaves were analyzed using GC-MS and UPLC-Q-Orbitrap HRMS techniques. A total of 78 volatile compounds and 66 non-volatile compounds were identified. The volatile compounds were predominantly terpenoids and aliphatic compounds, while the non-volatile compounds were primarily flavonoids and alkaloids. The branches contained 52 volatile compounds and 33 non-volatile compounds, whereas the leaves contained 48 volatile compounds and 40 non-volatile compounds. The antioxidant activities of the methanol extracts from *Z. myriacanthum* branches and leaves were evaluated using ABTS and DPPH free-radical-scavenging assays, both of which demonstrated certain antioxidant activity. The methanol extract of leaves demonstrated significantly higher antioxidant activity compared to that of the branches, possibly due to the higher presence of flavonoids and phenols in the leaves, with IC_50_ values of 7.12 ± 0.257 μg/mL and 1.22 × 10^2^ ± 5.01 μg/mL for ABTS and DPPH, respectively. These findings enhance our understanding of the chemical composition and antioxidant potential of *Z. myriacanthum*. The plant holds promise as a natural source of antioxidants for applications in pharmaceuticals, cosmetics, and functional foods. Further research can explore its broader biological activities and potential applications.

## 1. Introduction

*Zanthoxylum myriacanthum* Wall. ex Hook. f., a member of the Rutaceae family and the *Zanthoxylum* genus, is widely distributed in the southern and southwestern regions of China, as well as in tropical areas of Vietnam, Myanmar, India, and other Southeast Asian countries. The traditional use of the root bark, stem bark, and young leaves of this plant as herbal medicines for various ailments, including trauma, pediatric hernia, snake bites, ulcers, rheumatism, and pain, has been well documented [1,2]. *Z. myriacanthum* comprises an original variety and a variant, namely *Z. myriacanthum* var. *myriacanthum* and *Z. myriacanthum* var. *pubescens*, respectively [1]. *Z. myriacanthum* var. *pubescens*, commonly known as “Maqian,” is extensively employed as a food flavoring agent in China. The plant exhibits branches, leaflets, and fruits that emanate a distinctive and potent aroma due to the abundance of small oil glands [3]. Existing research has predominantly focused on the chemical composition and biological properties of the essential oil derived from *Z. myriacanthum* fruits. These fruits are characterized by a high essential oil content, accounting for approximately 4% of their dry weight [4,5]. The essential oil obtained from *Z. myriacanthum* fruits is in high demand across various industries, including food flavoring, traditional medicine, perfumery, and pharmaceuticals [5]. Previous studies have identified the major constituents of *Z. myriacanthum* essential oil as primarily limonene (67.06%), *α*-pinene (6.49%), *β*-myrcene (3.87%), and linalool (2.96%) [6]. Importantly, essential oil is also present in *Z. myriacanthum* seed coats, seeds, and whole fruits, with limonene being the primary chemical constituent, constituting 46.0%, 69.9%, and 42.8% of their respective compositions [7]. Extensive studies have demonstrated the anti-inflammatory [8], antiviral [9], and antimicrobial activities of *Z. myriacanthum* essential oil [6].

In contrast, limited attention has been given to the chemical constituents and biological activities of the original variety, *Z. myriacanthum* var. *myriacanthum*. Only a few studies have reported on the insecticidal activity of its volatile components. A recent study conducted an analysis of the essential oils derived from *Z. myriacanthum* fruit using GC-MS. The study found that dl-limonene accounted for 29.75% and sabinene for 9.76% of the essential oil composition. In the dichloromethane extract, the main components were identified as limonene (40.70%) and sabinene (16.60%). The fruit extract of *Z. myriacanthum* demonstrated insecticidal and repellent activities against two species of spider mites [10]. Moreover, the essential oil extracted from *Z. myriacanthum* exhibited insecticidal and repellent effects on three different pests, namely *Tribolium castaneum*, *Lasioderma serricorne*, and *Liposcelis bostrychophila*. These findings indicate that *Z. myriacanthum* essential oil has potential as a natural insecticide and repellent, offering valuable applications in the management of stored pests [11]. The non-volatile components of *Z. myriacanthum* have received limited attention, with only early literature reporting the isolation and identification of phenantridine alkaloids in *Z. myriacanthum* [12,13].

Therefore, the primary objective of this study was to explore the chemical composition and antioxidant activities of the branches and leaves of *Z. myriacanthum* var. *myriacanthum*, which have not been extensively investigated thus far. To achieve this goal, we employed GC-MS and UPLC-Q-Orbitrap HRMS techniques for the analysis of volatile and non-volatile components, respectively. Furthermore, the antioxidant potential of methanol extracts was assessed through DPPH and ABTS radical scavenging assays. These investigations contribute to a better understanding of the chemical constituents and potential health benefits of *Z. myriacanthum*. In conclusion, our study sheds light on the previously unexplored branches and leaves of *Z. myriacanthum*, providing valuable insights into their chemical composition and antioxidant activities. These findings pave the way for further research and the development of novel applications in the fields of medicine, functional foods, and natural product-based antioxidants. Moreover, they contribute to the overall knowledge and rational utilization of *Z. myriacanthum*.

## 2. Results

### 2.1. GC-MS Analysis of Volatile Components from Branches and Leaves of Z. myriacanthum

GC-MS analysis of the volatile components extracted from the branches and leaves of *Z. myriacanthum* led to the identification of 78 compounds, accounting for 82.91% and 87.79% of the total oil content, respectively. These compounds included 45 terpenoids, 29 aliphatic compounds, and 4 aromatic compounds. Among the volatile oil extracts from the branches, the highest proportions were observed for bicyclo[3.1.0]hexane (20.65%), terpinen-4-ol (13.34%), and *γ*-Terpinene (5.03%), all of which belong to the terpenoid group. Similarly, in the leaves, the highest proportions were found for D-Limonene (23.42%), caryophyllene (9.74%), and terpinen-4-ol (7.97%), also belonging to the terpenoid group. Detailed information on these volatile compounds found in the branches and leaves of *Z. myriacanthum* is provided in Table 1. Furthermore, the GC-MS chromatograms of volatile components extracted from the branches and leaves of *Z. myriacanthum* are shown in Figures S1 and S2 of the Supplementary Materials.

### 2.2. UPLC-Q-Orbitrap HRMS Analysis of Non-Volatile Components from Branches and Leaves of Z. myriacanthum

A comprehensive analysis using UPLC-Q-Orbitrap HRMS revealed the presence of 66 non-volatile components in the methanol extract of *Z. myriacanthum* branches and leaves. These compounds encompassed a variety of classes, including 25 flavonoids, 17 alkaloids, 9 fatty acids, 4 phenols, 3 phenylpropanoids, 3 esters, and 5 other compounds. For detailed information on these compounds, please refer to Table 2. The UPLC-Q-Orbitrap HRMS chromatograms illustrating the methanol extracts of *Z. myriacanthum* branches and leaves are presented in Figures S3–S6 of the Supplementary Materials.

### 2.3. Comparison of Constituents of Branches and Leaves of Z. myriacanthum

#### 2.3.1. Comparison of Volatile Components

The comparison of volatile components between the leaves and branches of *Z. myriacanthum* reveals a combination of shared and distinct compounds. Table 1 offers a comprehensive summary of the chemical composition, and peak-area normalization enables the calculation of relative mass fractions. The leaves contain a total of 48 components: 12 aliphatic compounds (2, 10, 14, 19, 23, 36, 37, 38, 42, 51, 52 and 62), 3 aromatic compounds (4, 56 and 67), and 33 terpenoids (6, 8, 11, 13, 16, 22, 25, 26, 27, 28, 29, 30, 31, 34, 43, 44, 46, 47, 49, 54, 58, 59, 60, 64, 65, 66, 68, 69, 70, 72, 73 and 75). The branches contain a total of 52 components: 26 aliphatic compounds (1, 2, 3, 7, 10, 12, 14, 18, 21, 23, 24, 32, 35, 38, 39, 40, 41, 42, 45, 48, 51, 52, 55, 62, 77 and 78), 3 aromatic compounds (4, 33 and 56), and 23 terpenoids (5, 6, 8, 9, 11, 13, 15, 17, 20, 22, 25, 26, 28, 31, 46, 47, 50, 53, 57, 61, 63, 71 and 74). A comparison of the volatile components allows for the identification of both shared and distinctive compounds. The shared volatile component consists of 9 aliphatic compounds, namely 2,4-dimethylheptane (2), *β*-myrcene (10), 4-methyldecane (14), *β*-linalool (23), tetradecane (38), 2,6,11-trimethyldodecane (42), 2,6,10-trimethyltetradecane (51), heptadecane (52), and 1,6,10-dodecatrien-3-ol (62). In addition, there are 2 aromatic compounds, styrene (4) and 2,4-di-tert-butylphenol (56), and 11 terpenes: *α*-pinene (6), bicyclo[3.1.0]hexane (8), *α*-phellandrene (11), *α*-terpinene (13), *α*-terpinolen (22), *trans*-*p*-menth-2-en-1-ol (25), terpinen-4-ol (26), *α*-terpineol (28), piperitol (31), caryophyllene (46), and *α*-caryophyllene (47). The structures of these compounds can be found in Figure S7 of the Supplementary Materials. These compounds are present in both the leaves and branches, indicating their ubiquity within the plant. Additionally, each part contains specific volatile components that are unique to it. The leaves exhibit 22 exclusive terpenoids, 3 exclusive aliphatic compounds and 1 exclusive aromatic compound, while the branches possess 17 unique aliphatic compounds, 12 unique terpenoids and 1 unique aromatic compound.

This comprehensive analysis unveils both the shared and distinct volatile components present in the leaves and branches of *Z. myriacanthum*. Further investigation of these compounds will advance our understanding of the plant’s chemical profile and its potential applications.

#### 2.3.2. Comparison of Non-Volatile Components

The comparison of methanol extract components in *Z. myriacanthum* revealed a predominant abundance of flavonoids and alkaloids, accompanied by a lesser amount of alkaloids, amino acids, phenylpropanoids, and terpenes (Table 2). Specifically, the analysis of leaves identified 40 components, including 21 flavonoids (1, 9, 11, 12, 14, 15, 17, 19, 20, 21, 22, 24, 26, 27, 29, 32, 34, 36, 40, 41 and 48), 4 alkaloids (3, 5, 23, 58), 7 fatty acids (4, 7, 8, 43, 45, 50 and 53), 3 phenols (6, 31 and 35), 2 phenylpropanoids (10 and 13), 1 ester (16), and several other compounds (46). A total of 33 non-volatile components were identified in the branches, including 6 flavonoids (28, 32, 33, 39, 41, 54), 15 alkaloids (2, 5, 23, 25, 37, 38, 42, 47, 49, 51, 52, 59, 61, 63 and 65), 4 fatty acids (7, 44, 45 and 57), 1 phenylpropanoid (30), 3 esters (16, 56 and 66), and several other compounds (19, 55, 60 and 64). The non-volatile components identified in the methanol extracts of *Z. myriacanthum* branches and leaves exhibited significant variations. Among these components, only 7 were shared between them. These components include kynurenic acid (5), d-(−)-quinic acid (7), 3-(benzoyloxy)-2-hydroxypropyl-*β*-d-glucopyranosiduronic acid (16), *N*-acetyltryptophan (23), diosmin (32), didymin (41), and (9*Z*)-5,8,11-trihydroxy-9-octadecenoic acid (45). The structures of these compounds can be found in Figure S8 of the supplementary materials. This comprehensive analysis of methanol extract components offers valuable insights into the overlapping and distinctive chemical profiles of *Z. myriacanthum* leaves and branches. Further investigations into these compounds will contribute to a more profound understanding of their potential biological activities and therapeutic applications.

### 2.4. Antioxidant Activity

**Figure 1:**
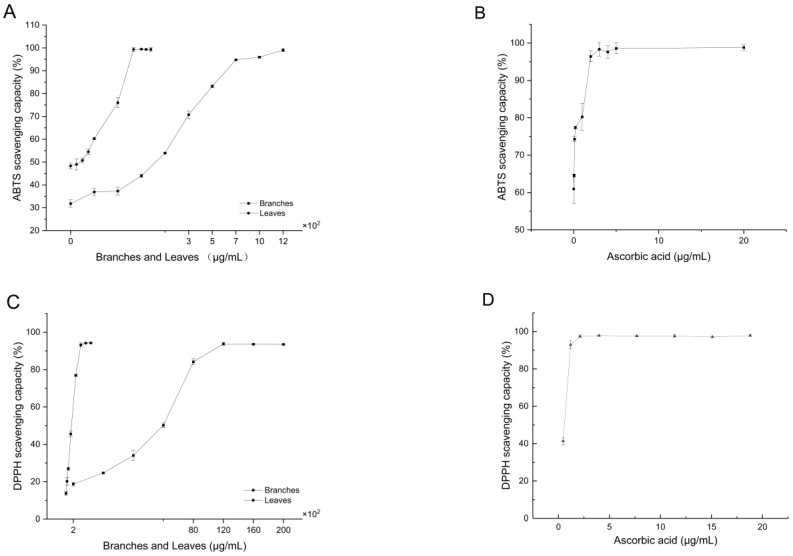
The radical scavenging capacity of methanol extracts of branches and leaves of *Z. myriacanthum* compared with that of ascorbic acid. (**A**) ABTS scavenging capacity of leaves and branches, (**B**) ABTS scavenging capacity of ascorbic acid control, (**C**) DPPH scavenging capacity of leaves and branches, and (**D**) ABTS scavenging capacity of ascorbic acid control. Each data point represents the mean ± SD of three replicates (N = 3) at different concentrations.

In this study, the branches and leaves of *Z. myriacanthum* were assessed using ABTS and DPPH radical scavenging assays, respectively, and compared to ascorbic acid standards, and Figure 1 displayed the outcomes. The results obtained from the ABTS and DPPH antioxidant assays demonstrated that the leaves of *Z. myriacanthum* (ABTS: 7.12 ± 0.257 μg/mL, DPPH: 1.22 × 10^2^ ± 5.01 μg/mL) exhibited superior antioxidant activity compared to that of the branches (ABTS: 5.54 × 10^1^ ± 4.34 μg/mL, DPPH: 2.93 × 10^3^ ± 8.43 × 10^1^ μg/mL), as indicated in Table 3. However, both were less potent than the antioxidant activity of ascorbic acid (ABTS: 6.12 × 10^−3^ ± 1.76 × 10^−3^ μg/mL, DPPH: 8.12 ± 4.20 × 10^−2^ μg/mL).

## 3. Discussion

The present study aimed to analyze the volatile and methanol-extract components of *Z. myriacanthum* branches and leaves and evaluate their antioxidant activity. The findings shed light on the chemical composition and potential applications of this plant.

In the analysis of volatile components, GC-MS analysis identified a total of 78 compounds in the branches and leaves, with 45 terpenoids, 29 aliphatic compounds, and 4 aromatic compounds. The major volatile components differed between the branches and leaves, with bicyclo[3.1.0]hexane, terpinen-4-ol, and *γ*-Terpinene being predominant in the branches, and D-Limonene, caryophyllene, and terpinen-4-ol being major components in the leaves. The comparison between branches and leaves identified shared volatile compounds, including aliphatic compounds, aromatic compounds, and terpenoids. Additionally, each part had unique volatile components, with the leaves containing 22 exclusive terpenoids, 3 exclusive aliphatic compounds, and 1 exclusive aromatic compound, while the branches had 17 unique aliphatic compounds, 12 unique terpenoids, and 1 unique aromatic compound. Using GC-MS technology, previous studies have also identified terpenoids, such as limonene and sabinene, in *Z. myriacanthum*, highlighting their anti-insect activity. Similarly, this study identified terpenoids like D-limonene, as well as aliphatic and aromatic compounds [6]. The volatile components terpinen-4-ol, *γ*-terpinene, and D-limonene found both in branches and leaves of *Z. myriacanthum* have demonstrated anti-cancer, anti-inflammatory, and immunomodulatory activities in previous research. Terpinen-4-ol has been shown to enhance the effects of various chemotherapeutic and biological agents, potentially acting as an anticancer agent [67]. *γ*-Terpinene has demonstrated anti-inflammatory properties by reducing paw edema and inhibiting neutrophil migration and the production of pro-inflammatory cytokines [68]. D-limonene and its metabolites have been found to modulate the immune response by inhibiting the production of certain cytokines and inducing T lymphocyte death [69].

In the analysis of methanol extracts, a total of 66 compounds were identified in the branches and leaves of *Z. myriacanthum*, belonging to various classes such as flavonoids, alkaloids, fatty acids, phenols, phenylpropanoids, esters, and other compounds. The comparison of chemical composition between branches and leaves revealed both shared and unique compounds. Only a few components were shared between the two parts, while the majority of compounds were exclusive to either branches or leaves. *Z. myriacanthum* is rich in flavonoids, which have garnered attention due to their medicinal properties and effectiveness [70]. Diosmin, identified in the methanol extract of branches and leaves, possesses antioxidant activity. Administration of diosmin has been shown to reduce oxidative stress markers significantly [71]. Previous studies have indicated the antioxidant potential of *Z. myriacanthum* fruits. The essential oil derived from the fruits exhibited strong renal protective effects by alleviating oxidative stress in diabetic mice [8]. Additionally, the use of the supercritical fluid extraction (SFE) method to obtain extracts from *Z. myriacanthum* fruits showed significant antioxidant activity in DPPH and ABTS assays, with IC_50_ values of 26.06 and 19.90 μg/mL, respectively [72]. In this study, the antioxidant activity of methanol extracts from *Z. myriacanthum* branches and leaves was evaluated. The results revealed the antioxidant potential of the methanol extracts, particularly the leaf extract. This disparity may be attributed to the presence of unique active ingredients in the leaves, including 19 flavonoids and 4 phenols. The IC_50_ values of ABTS and DPPH assays were found to be 7.12 ± 0.257 and 1.22 × 10^2^ ± 5.01 μg/mL, respectively. It is noteworthy that these concentrations represent the dry mass powder per 1 mL of solvent, indicating better antioxidant activity compared to the previously reported DPPH and ABTS antioxidant activities of *Z. myriacanthum*.

In conclusion, GC-MS and UPLC-Q-Orbitrap HRMS analyses were employed to investigate the volatile oil and methanol extracts of *Z. myriacanthum* branches and leaves, revealing a diverse array of compounds. The comparison between the two parts highlighted both shared and distinctive components, contributing to a better understanding of the plant’s chemical profile. Furthermore, the antioxidant activity of *Z. myriacanthum* leaves and branches was demonstrated, emphasizing their potential as a natural source of antioxidants. These findings provide a foundation for future studies exploring the biological activities and potential applications of *Z. myriacanthum* in various fields, including pharmaceuticals, cosmetics, and functional foods.

## 4. Materials and Methods

### 4.1. Plant Material and Extraction

#### 4.1.1. Plant Material

*Z. myriacanthum* was collected from Yunfu, China in April 2023. The plant materials were identified by Dr. Xinger Ye, College of Traditional Chinese Medicine Resources, Guangdong Pharmaceutical University.

#### 4.1.2. Hydro-Distillation of Volatile Components and Preparation of Methanol Extracts

Fresh branches and leaves of *Z. myriacanthum* weighing 50 g were finely minced and placed in 500 mL of distilled water. The mixture was then subjected to hydro-distillation using a Clevenger-type apparatus for 4 h. The resulting volatile oil was extracted using *n*-hexane, dried with anhydrous sodium sulfate, and stored in a brown glass bottle at a temperature of 4–6 °C until analysis.

For the preparation of methanol extracts, 1.0 g of dried branches and leaf powder from *Z. myriacanthum* was weighed and mixed with 50 mL of methanol. The mixture was thoroughly blended and subjected to ultrasound-assisted extraction for 30 min at a temperature of 50 °C. Subsequently, the mixture was cooled to room temperature, and any weight loss was compensated for by adding methanol. A 10 mL aliquot of the supernatant was transferred to a centrifuge tube and centrifuged at a speed of 4500 r/min for 15 min. After centrifugation, 200 μL of the supernatant was taken, diluted to 1 mL with methanol, thoroughly mixed, and filtered through a 0.22 μm filter.

### 4.2. The Main Chemicals and Reagents

UPLC-Q-Orbitrap HRMS analysis was conducted using the Vanquish Flex UHPLC system and Orbitrap Exploris 120 quadrupole electrostatic field orbital well high-resolution mass spectrometer from Thermo Fisher Scientific (Waltham, MA, USA). Gas chromatography-mass spectrometry (GC-MS) analysis was performed using the Agilent 8890 GC System-5977B GC/MSD from Agilent Technologies (Santa Clara, CA, USA). The absorbance measurements were recorded using the Agilent Synergy H1 multifunction microplate reader (Agilent Technologies, USA). The DFY-300C Swing Crusher was obtained from Wenling Linda Machinery Co., Ltd. (Wenling, China). The ATY 1/24 million balance was supplied by Shimadzu Enterprise Management (China) Co., Ltd. (Beijing, China). The KQ-500DE Desktop CNC Ultrasonic Cleaner was acquired from Dongguan Keqiao Ultrasonic Equipment Co., Ltd. (Dongguan, China). The following chemicals were used: *n*-hexane (GC-grade), 1,1-diphenyl-2-trinitrophenylhydrazine (DPPH), and 2,2′-diazo-bis(3-ethylbenzothiazole-6-sulfonic acid) (ABTS) (purity > 98%) from Alsan Biotechnology Co., Guangzhou, China; ascorbic acid from Guangzhou Chemical Reagent Factory, Guangzhou, China; analytical grade methanol purchased from Da Mao Chemical Reagent Co., Ltd., Tianjin, China; chromatography-grade acetonitrile obtained from Honeywell Trading (Shanghai) Co., Ltd., Shanghai, China; and distilled water from Watsons, Hong Kong, China.

### 4.3. GC-MS Analysis

#### 4.3.1. Instrumentation and Conditions

The substances in the samples were separated using an Agilent 8890 series gas chromatograph, and these substances were quantified and identified using an Agilent 5977B series mass spectrometer. The chromatographic conditions of the Agilent 8890 were as follows: The chromatographic column used was an Agilent 19091S-433UI: 0263036H column with dimensions of 30 m × 250 µm × 0.25 µm. The sample injection volume was 1 μL, and the injection port temperature was set to 250 °C. The temperature program employed was as follows: starting at 50 °C for 0 min, then ramping to 140 °C at a rate of 6 °C/min, followed by an increase to 160 °C at a rate of 2.5 °C/min, and finally reaching 240 °C at a rate of 12 °C/min, with a hold time of 5 min. The carrier gas used was high-purity helium with a flow rate of 1 mL/min, and the injection port was operated in the undivided mode. The GC column was directly connected to an Agilent 5977B series mass selective detector with an ion source for mass spectrometry analysis. The electron ionization (EI) source was utilized for ionization, with the analyte being ionized at 70 eV and 230 °C in the ion source. The scanning mass range was set from 50 to 550.

#### 4.3.2. Data Analysis and Identification of Compounds

The raw data files were imported into Qualitative Analysis 10.0 software for further analysis. Peak integration and extraction of mass spectra were conducted using this software. The extracted mass spectra were compared against the NIST standard library for identification. Additionally, peak extraction and alignment were performed on the raw data. The identification of compounds was accomplished by combining relevant literature and utilizing online databases.

### 4.4. UPLC-Q-Orbitrap HRMS Analysis

#### 4.4.1. Instrumentation and Conditions

The UPLC-Q-Orbitrap HRMS analysis was conducted using the Vanquish Flex UHPLC system coupled with the Orbitrap Exploris 120 quadrupole electrostatic field orbital well high-resolution mass spectrometer. A Hypersil GOLD C_18_ analytical column (100 mm × 2.1 mm, 5 µm) from Thermo Fisher Scientific was employed for separation at a temperature of 35 °C. The mobile phase consisted of acetonitrile (A) and water/formic acid 0.1% *v*/*v* (B), and a gradient elution method was applied at a flow rate of 0.3 mL/min. The gradient conditions were as follows: 95% to 80% B from 0 to 5 min, 80% to 75% B from 5 to 8 min, 75% to 5% B from 8 to 20 min, 5% B at 20–22 min, 5% to 95% B from 22 to 22.001 min, and finally 95% B from 22.001 to 25 min. The sample injection volume was 2.0 µL.

The mass spectrometer operated in both positive and negative ion modes. The MS detection parameters were optimized as follows: spray voltage of +3.5 kV for positive ion mode and −2.8 kV for negative ion mode, ion transfer tube temperature of 325 °C, sheath gas at 50 arbitrary units, AUX gas at 8 arbitrary units, sweep gas at 1 arbitrary unit, vaporizer temperature at 350 °C, RF Lens at 70%, scan range of *m*/*z* 100–1500, and a resolution of 60,000 (MS) and 15,000 (MS^2^). Stepped normalized collision energy (NCE) of 20%, 40%, and 60% was applied, and Orbitrap mass calibration was performed once a week to ensure accurate mass measurement.

#### 4.4.2. Data Analysis and Identification of Compounds

The raw data files were imported into the Compound Discoverer 3.3 software for further analysis. Peak extraction and alignment of the original data were performed using the compound identification-method template. The secondary fragment spectra were matched against the mzCloud and mzVault databases. The matching results underwent filtering based on the following criteria: elimination of blank background ions, quality deviation of primary and secondary levels within 5 ppm, and a minimum mzCloud or mzVault score of 80. The filtered ions were then compared with the compound information in the database. Further analysis of the compounds was conducted by considering relevant literature and utilizing online databases such as PubChem, CNKI, and PubMed.

### 4.5. Antioxidant Activity

#### 4.5.1. ABTS Radical Scavenging Assay

The 2,2′-azino-bis-3-ethylbenzthiazoline-6-sulphonic acid (ABTS) solution was prepared in advance. One milliliter of extracts from branches and leaves was mixed with one milliliter of ABTS solution. The mixture was then allowed to react at room temperature for 30 min in the dark. The absorbance at 734 nm was recorded using a microplate reader from Agilent [73,74]. The IC_50_ value represents the concentration of the phenolic extract required to scavenge 50% of the ABTS radicals. The ABTS radical scavenging capacity was determined using Equation (1):

(1) $$\mathrm{ABTS}-\mathrm{scavenging}\;\mathrm{activity}\;(\%)=(\;1-\frac{A_{1}-A_{2}}{A_{0}}\;)\times 100\%$$

where A_0_ represents the absorbance of the control (methanol replacing the sample), A_1_ represents the absorbance of the sample, and A_2_ represents the absorbance of the sample and ethanol without ABTS.

#### 4.5.2. DPPH Radical Scavenging Assay

In the experiment, a 1,1-diphenyl-2-trinitrophenylhydrazine (DPPH) solution with a concentration of 0.3 mmol/L was prepared. Then, 100 μL of the DPPH solution was added to each well of a 96-well plate. Subsequently, 100 μL of different concentrations of extracts from branches and leaves were added to the wells. The reaction took place in a dark environment at room temperature for 30 min. After the reaction, the absorbance of the samples was measured at 517 nm using a microplate reader (Agilent, Shanghai, China). The IC_50_ value represents the concentration of the phenolic extract required to scavenge 50% of the DPPH radicals. The DPPH radical scavenging capacity was calculated using the following Equation (2):

(2) $$\mathrm{DPPH}-\mathrm{scavenging}\;\mathrm{activity}\;(\%)=(\;1-\frac{A_{1}-A_{2}}{A_{0}}\;)\times 100\%$$

where A_0_ represents the absorbance of the control (methanol replacing the sample), A_1_ represents the absorbance of the sample, and A_2_ represents the absorbance of the sample and ethanol without DPPH.

#### 4.5.3. Statistical Analysis

All experiments were repeated three times, and the results were expressed as mean ± SD.

## 5. Conclusions

This study comprehensively analyzed the chemical composition and antioxidant activities of *Z*. *myriacanthum* branches and leaves using GC-MS and UPLC-Q-Orbitrap HRMS techniques. The results revealed a rich diversity of volatile and non-volatile compounds in both parts of the plant. The volatile compounds mainly consisted of terpenoids and aliphatic compounds, exhibiting distinct differences between the two plant parts. The analysis of methanol extracts identified various classes of compounds, including flavonoids, alkaloids, fatty acids, and phenols. The leaves showed significantly higher antioxidant activity compared to the branches, attributed to the presence of unique active ingredients, such as flavonoids and phenols. These findings underscore the plant’s potential as a natural antioxidant source for pharmaceuticals, cosmetics, and functional foods. Further research is warranted to explore its broader biological activities and potential applications in various industries. Overall, this study contributes to a deeper understanding of *Z. myriacanthum*’s therapeutic properties and encourages further investigation into its diverse potential.

## Figures and Tables

**Table 1:** Identified constituents and composition of volatile components in *Z. myriacanthum* branches and leaves by GC-MS.

No.	RT/min		*m*/*z*	Compound	Molecular Formula	Compound Types	% Composition	
	Branches	Leaves					Branches	Leaves
1	3.431	–	114.1	heptane	C_8_H_18_	aliphatic compounds	0.24	–
2	4.810	4.833	128.1	2,4-dimethylheptane	C_9_H_20_	aliphatic compounds	1.30	0.63
3	6.194	–	128.1	4-methyloctane	C_9_H_20_	aliphatic compounds	0.36	–
4	7.098	7.110	104.0	styrene	C_8_H_8_	aromatic compounds	2.12	1.00
5	8.346	–	136.1	*β*-thujene	C_10_H_16_	terpenoids	0.44	–
6	8.552	8.558	136.1	*α*-pinene	C_10_H_16_	terpenoids	5.01	0.84
7	9.599	–	142.1	2-methylnonane	C_10_H_22_	aliphatic compounds	0.23	–
8	9.879	9.879	136.1	bicyclo[3.1.0]hexane	C_10_H_16_	terpenoids	20.65	0.68
9	9.959	–	136.1	*β*-pinene	C_10_H_16_	terpenoids	0.79	–
10	10.469	10.474	136.1	*β*-myrcene	C_10_H_16_	aliphatic compounds	0.77	2.19
11	10.846	10.852	136.1	*α*-phellandrene	C_10_H_16_	terpenoids	0.51	0.59
12	11.184	–	156.2	2,5-dimethylnonane	C_11_H_24_	aliphatic compounds	0.30	–
13	11.235	11.236	136.1	*α*-terpinene	C_10_H_16_	terpenoids	2.06	0.29
14	11.441	11.361	156.2	4-methyldecane	C_11_H_24_	aliphatic compounds	0.97	0.48
15	11.607	–	136.1	pseudolimonen	C_10_H_16_	terpenoids	2.06	1.16
16	–	11.630	136.1	d-limonene	C_10_H_16_	terpenoids	–	23.42
17	11.682	–	154.1	eucalyptol	C_10_H_18_O	terpenoids	1.64	–
18	12.220	–	136.1	*β*-*cis*-ocimene	C_10_H_16_	aliphatic compounds	0.58	–
19	–	12.220	136.1	*β*-ocimene	C_10_H_16_	aliphatic compounds	–	1.39
20	12.511	–	136.1	*γ*-terpinene	C_10_H_16_	terpenoids	5.03	–
21	12.649	–	155.0	2,4,6-trimethyldecane	C_13_H_28_	aliphatic compounds	0.51	–
22	13.364	13.370	136.1	*α*-terpinolen	C_10_H_16_	terpenoids	0.73	0.57
23	13.696	13.707	156.1	*β*-linalool	C_10_H_18_O	aliphatic compounds	0.40	5.69
24	13.759	–	155.1	undecane	C_11_H_24_	aliphatic compounds	0.43	–
25	14.285	14.285	154.1	*trans*-*p*-menth-2-en-1-ol	C_10_H_18_O	terpenoids	0.65	1.64
26	15.796	15.802	154.1	terpinen-4-ol	C_10_H_18_O	terpenoids	13.34	7.97
27	–	16.031	150.1	2,6a-methano-6a*H*-indeno[4,5-b]oxirene	C_10_H_14_O	terpenoids	–	0.31
28	16.128	16.134	136.1	*α*-terpineol	C_10_H_18_O	terpenoids	1.93	2.07
29	–	16.254	154.1	*trans*-piperitol	C_10_H_18_O	terpenoids	–	0.37
30	–	16.443	154.1	bicyclo[2.2.1]heptan-2-ol	C_10_H_18_O	terpenoids	–	0.50
31	16.563	16.254	154.1	piperitol	C_10_H_18_O	terpenoids	0.30	0.87
32	16.689	–	184.2	2,6-dimethylundecane	C_13_H_28_	aliphatic compounds	0.29	–
33	16.740	–	134.0	isoxylaldehyde	C_9_H_10_O	aromatic compounds	0.34	–
34	–	16.832	152.1	*cis*-Carveol	C_10_H_16_O	terpenoids	–	0.35
35	16.889	–	184.2	4,8-dimethylundecane	C_13_H_28_	aliphatic compounds	0.26	–
36	–	17.055	154.1	2,6-octadien-1-ol	C_10_H_18_O	aliphatic compounds	–	0.27
37	–	17.684	154.1	2,7-dimethyl-2,6-octadien-1-ol	C_10_H_18_O	aliphatic compounds	–	0.89
38	17.907	17.907	198.2	tetradecane	C_14_H_30_	aliphatic compounds	0.76	0.35
39	18.497	–	169.1	pentadecane	C_15_H_32_	aliphatic compounds	0.39	–
40	18.623	–	198.2	4,6-dimethyldodecane	C_14_H_30_	aliphatic compounds	0.31	–
41	18.806	–	198.2	2,3,5,8-tetramethyldecane	C_14_H_30_	aliphatic compounds	0.24	–
42	19.361	19.361	212.2	2,6,11-trimethyldodecane	C_15_H_32_	aliphatic compounds	0.45	0.56
43	–	19.967	204.1	*α*-cubebene	C_15_H_24_	terpenoids	–	0.30
44	–	20.591	204.1	copaene	C_15_H_24_	terpenoids	–	0.49
45	21.587	–	183.2	nonadecane	C_19_H_40_	aliphatic compounds	0.29	–
46	21.667	21.678	204.2	caryophyllene	C_15_H_24_	terpenoids	4.19	9.74
47	22.531	22.531	204.2	*α*-caryophyllene	C_15_H_24_	terpenoids	0.85	1.87
48	22.588	–	226.2	2,6,10-trimethyltridecane	C_16_H_34_	aliphatic compounds	0.46	–
49	–	23.258	204.1	*β*-cubebene	C_15_H_24_	terpenoids	–	0.98
50	23.263	–	204.2	*β*-copaene	C_15_H_24_	terpenoids	1.80	–
51	23.349	23.349	240.2	2,6,10-trimethyltetradecane	C_17_H_36_	aliphatic compounds	0.43	0.26
52	23.515	23.515	281.0	heptadecane	C_21_H_44_	aliphatic compounds	1.12	0.59
53	23.675	–	204.1	elixene	C_15_H_24_	terpenoids	0.21	–
54	–	23.681	204.1	*γ*-elemene	C_15_H_24_	terpenoids	–	0.49
55	23.887	–	204.1	α-farnesene	C_15_H_24_	aliphatic compounds	0.32	–
56	23.967	23.973	206.2	2,4-di-tert-butylphenol	C_14_H_22_O	aromatic compounds	1.08	0.75
57	24.396	–	204.2	*β*-cadinene	C_15_H_24_	terpenoids	0.47	–
58	–	24.396	204.1	cadina-1(10),4-diene	C_15_H_24_	terpenoids	–	1.26
59	–	24.906	220.1	*α*-copaen-11-ol	C_15_H_24_O	terpenoids	–	0.31
60	–	25.134	222.1	cyclohexanemethanol	C_15_H_26_O	terpenoids	–	2.24
61	25.420	–	221.0	(−)-globulol	C_15_H_26_O	terpenoids	0.26	–
62	25.495	25.501	222.1	1,6,10-dodecatrien-3-ol	C_15_H_26_O	aliphatic compounds	1.90	3.81
63	26.210	–	222.1	globulol	C_15_H_26_O	terpenoids	0.37	–
64	–	26.210	222.1	epiglobulol	C_15_H_26_O	terpenoids	–	0.48
65	–	26.611	222.1	guaiol	C_15_H_26_O	terpenoids	–	1.82
66	–	26.822	222.1	1*H*-cycloprop[e]azulen-4-ol	C_15_H_26_O	terpenoids	–	0.48
67	–	27	340.2	2,2′-Methylenebis(6-tert-butyl-p-cresol)	C_23_H_32_O_2_	aromatic compounds	–	0.26
68	–	27.566	222.1	1,10-Di-epi-Cubenol	C_15_H_26_O	terpenoids	–	0.44
69	–	27.675	222.1	(+)-*γ*-Eudesmol	C_15_H_26_O	terpenoids	–	0.76
70	–	28.007	222.1	*τ*-Cadinol	C_15_H_26_O	terpenoids	–	0.69
71	28.115	–	222.1	cubebol	C_15_H_26_O	terpenoids	0.30	–
72	–	28.259	222.1	2-naphthalenemethanol	C_15_H_26_O	terpenoids	–	0.4
73	–	28.362	222.1	maaliol	C_15_H_26_O	terpenoids	–	1.21
74	28.373	–	222.1	epi-*α*-Muurolol	C_15_H_26_O	terpenoids	1.15	–
75	–	28.728	222.1	5-azulenemethanol	C_15_H_26_O	terpenoids	–	1.13
76	–	29.643	220.1	aromadendrene oxide-(1)	C_15_H_24_O	terpenoids	–	1.95
77	29.666	–	380.4	heptacosane	C_27_H_56_	aliphatic compounds	1.01	–
78	31.840	–	294.2	(3*E*,12*Z*)-1,3,12-nonadecatriene-5,14-diol	C_19_H_34_O_2_	aliphatic compounds	0.31	–
Total							82.91	87.79

–: not detected.

**Table 2:** Compounds identified in the methanol extract of *Z. myriacanthum* branches and leaves by UPLC-Q-Orbitrap HRMS.

No.	RT/min		Compound	Molecular Formula	Error/ppm	*m*/*z*	Ion Mode	Compound Types	References
	Branches	Leaves							
1	–	2.260	epigallocatechin	C_15_H_14_O_7_	−1.48	305.06622	[M − H]^−^	flavonoids	[14]
2	2.960	–	*trans*-3-indoleacrylic acid	C_11_H_9_NO_2_	−0.4	188.07054	[M + H]^+^	alkaloids	[15]
3	–	3.086	8-hydroxyquinoline	C_9_H_7_NO	0.34	146.06009	[M + H]^+^	alkaloids	[16]
4	–	3.092	indole-3-acrylic acid	C_11_H_9_NO_2_	−0.24	188.07056	[M + H]^+^	fatty acids	[17]
5	3.464	3.515	kynurenic acid	C_10_H_7_NO_3_	−0.18	190.04984	[M + H]^+^	alkaloids	[18]
6	–	4.086	catechin	C_15_H_14_O_6_	−0.47	289.07162	[M − H]^−^	phenols	[19]
7	4.249	4.236	d-(−)-quinic acid	C_7_H_12_O_6_	−0.34	191.05605	[M − H]^−^	fatty acids	[20]
8	–	4.408	*p*-coumaric acid glucoside	C_15_H_18_O_8_	−0.73	325.09265	[M − H]^−^	fatty acids	[21]
9	–	5.317	dihydromyricetin	C_15_H_12_O_8_	−0.53	319.04578	[M − H]^−^	flavonoids	[22]
10	–	5.399	4-[3-(3,4-Dihydroxyphenyl)acryloyloxy]-2,3-dihydroxy-2-methylbutyric acid	C_14_H_16_O_8_	−0.78	311.077	[M − H]^−^	phenylpropanoids	
11	–	5.575	myricetin	C_15_H_10_O_8_	0.44	319.04498	[M + H]^+^	flavonoids	[23]
12	–	5.622	orientin	C_21_H_20_O_11_	0.3	449.10797	[M + H]^+^	flavonoids	[24]
13	–	5.969	3-*O*-feruloylquinic acid	C_17_H_20_O_9_	−0.58	367.10324	[M − H]^−^	phenylpropanoids	[25]
14	–	5.986	cynaroside	C_21_H_20_O_11_	−0.8	447.09293	[M − H]^−^	flavonoids	[26]
15	–	6.012	myricetin-3-*O*-galactoside	C_21_H_20_O_13_	−1.17	479.08255	[M − H]^−^	flavonoids	[27]
16	6.051	6.046	3-(benzoyloxy)-2-hydroxypropyl-*β*-d-glucopyranosiduronic acid	C_16_H_20_O_10_	−1.02	371.09799	[M − H]^−^	esters	[28]
17	–	6.252	vitexin	C_21_H_20_O_10_	0.36	433.11307	[M + H]^+^	flavonoids	[29]
18	6.395	–	*N*-acetyl-d-phenylalanine	C_11_H_13_NO_3_	−0.77	206.08211	[M − H]^−^	amino acids	[30]
19	–	6.455	quercetin	C_15_H_10_O_7_	0.5	303.05008	[M + H]^+^	flavonoids	[31]
20	–	6.456	isoquercetin	C_21_H_20_O_12_	0.38	465.10291	[M + H]^+^	flavonoids	[29]
21	–	6.681	myricetin-3-xyloside	C_20_H_18_O_12_	−0.77	449.0722	[M − H]^−^	flavonoids	[32]
22	–	6.876	apigetrin	C_21_H_20_O_10_	−0.73	431.09805	[M − H]^−^	flavonoids	[33]
23	7.154	7.157	*N*-acetyltryptophan	C_13_H_14_N_2_O_3_	−0.42	245.09306	[M − H]^−^	alkaloids	[34]
24	–	7.162	sphaerobioside	C_27_H_30_O_14_	0.69	579.1712	[M + H]^+^	flavonoids	[35]
25	7.472	–	corydine	C_20_H_23_NO_4_	0.26	342.17007	[M + H]^+^	alkaloids	[36]
26	–	7.613	trifolin	C_21_H_20_O_11_	−0.6	447.09302	[M − H]^−^	flavonoids	[37]
27	–	7.628	avicularine	C_20_H_18_O_11_	−0.49	433.07742	[M − H]^−^	flavonoids	[38]
28	7.814	–	chromone	C_27_H_32_O_14_	−0.25	579.17163	[M − H]^−^	flavonoids	[39]
29	–	7.889	isorhoifolin	C_27_H_30_O_14_	−0.89	577.15576	[M − H]^−^	flavonoids	[40]
30	7.948	–	isochlorogenic acid A	C_25_H_24_O_12_	−0.46	515.11926	[M − H]^−^	phenylpropanoids	[41]
31	–	8.241	phloretin	C_15_H_14_O_5_	0	275.0914	[M + H]^+^	phenols	[42]
32	8.343	8.346	diosmin	C_28_H_32_O_15_	−1.66	607.16583	[M − H]^−^	flavonoids	[43]
33	8.475	–	hispidulin 4′-*O*-*β*-d-glucopyranoside	C_22_H_22_O_11_	1.03	463.12396	[M + H]^+^	flavonoids	[44]
34	–	8.485	neohesperidin	C_28_H_34_O_15_	−1.4	609.18164	[M − H]^−^	flavonoids	[45]
35	–	9.107	phloridzin	C_21_H_24_O_10_	−0.6	435.12939	[M − H]^−^	phenols	[46]
36	–	9.737	glycitein	C_16_H_12_O_5_	−0.13	285.07571	[M + H]^+^	flavonoids	[47]
37	10.057	–	paprazine	C_17_H_17_NO_3_	0.29	284.1282	[M + H]^+^	alkaloids	[48]
38	10.192	–	dihydrosanguinarine	C_20_H_15_NO_4_	0.48	334.10754	[M + H]^+^	alkaloids	[49]
39	10.891	–	biochanin A 7-*O*-rutinoside	C_28_H_32_O_14_	−0.36	637.17719	[M + FA − H]^−^	flavonoids	
40	–	10.891	acaciin	C_28_H_32_O_14_	−1.23	637.17664	[M + FA − H]^−^	flavonoids	[50]
41	11.254	11.065	didymin	C_28_H_34_O_14_	−0.2	593.1875	[M − H]^−^	flavonoids	[51]
42	11.670	–	*N*-(4-benzoylphenyl)propanamide	C_16_H_15_NO_2_	−0.01	254.11755	[M + H]^+^	alkaloids	
43	–	12.310	(11*E*,15*Z*)-9,10,13-trihydroxy-11,15-octadecadienoic acid	C_18_H_32_O_5_	−0.61	327.2175	[M − H]^−^	fatty acids	
44	12.317	–	(10*E*,15*Z*)-9,12,13-trihydroxy-10,15-octadecadienoic acid	C_18_H_32_O_5_	−0.23	327.21762	[M − H]^−^	fatty acids	[52]
45	12.848	12.833	(9*Z*)-5,8,11-trihydroxy-9-octadecenoic acid	C_18_H_34_O_5_	−0.4	329.23322	[M − H]^−^	fatty acids	
46	–	13.658	bis(4-ethylbenzylidene)sorbitol	C_24_H_30_O_6_	0.7	415.21176	[M + H]^+^	ethers	
47	13.938	–	*N*-[2-(4-methoxyphenyl)ethyl]-3-methyl-2-butenamide	C_14_H_19_NO_2_	−0.22	234.1488	[M + H]^+^	alkaloids	
48	–	14.631	5,7-dihydroxy-2-(4-hydroxyphenyl)-4-oxo-4h-chromen-3-yl 6-deoxy-3,4-bis-*O*-[(2*E*)-3-(4-hydroxyphenyl)-2-propenoyl]-α-l-mannopyranoside	C_39_H_32_O_14_	−0.07	723.17188	[M − H]^−^	flavonoids	
49	14.820	–	*N*-phenethyl-4-methoxybenzamide	C_16_H_17_NO_2_	−0.1	256.13318	[M + H]^+^	alkaloids	[53]
50	–	14.986	12-OPDA	C_18_H_28_O_3_	−0.04	293.21109	[M + H]^+^	fatty acids	[54]
51	15.279	–	dicyclohexylurea	C_13_H_24_N_2_O	−0.29	225.19608	[M + H]^+^	alkaloids	[55]
52	15.874	–	amphoteric L	C_19_H_38_N_2_O_3_	0.22	343.29559	[M + H]^+^	alkaloids	[56]
53	–	15.921	cis,cis-muconic acid	C_6_H_6_O_4_	0.01	141.01933	[M − H]^−^	fatty acids	[57]
54	16.527	–	isopongaflavone	C_21_H_18_O_4_	0.25	335.12787	[M + H]^+^	flavonoids	[58]
55	16.965	–	4-ethylbenzaldehyde	C_9_H_10_O	−0.21	135.08041	[M + H]^+^	aromatic aldehydes	[58]
56	16.975	–	4-ethoxy ethylbenzoate	C_11_H_14_O_3_	0.19	195.10161	[M + H]^+^	esters	[59]
57	17.105	–	coriolic acid	C_18_H_32_O_3_	−0.18	295.22782	[M − H]^−^	fatty acids	[60]
58	–	17.875	erucamide	C_22_H_43_NO	0.62	338.34195	[M + H]^+^	alkaloids	[61]
59	18.131	–	asperphenamate	C_32_H_30_N_2_O_4_	0.35	507.228	[M + H]^+^	alkaloids	[62]
60	18.668	–	(+)-isopetasol	C_15_H_22_O_2_	−0.03	235.16925	[M + H]^+^	terpenes	
61	19.659	–	kalecide	C_16_H_29_NO	0.07	252.23221	[M + H]^+^	alkaloids	[63]
62	–	19.925	2,2′-Methylenebis(4-methyl-6-tert-butylphenol)	C_23_H_32_O_2_	−1.38	339.23248	[M − H]^−^	phenols	[64]
63	20.329	–	linoleoyl ethanolamide	C_20_H_37_NO_2_	0.28	324.28979	[M + H]^+^	alkaloids	[65]
64	21.622	–	muscone	C_16_H_30_O	0.11	239.23697	[M + H]^+^	ketones	[66]
65	21.763	–	stearamide	C_18_H_37_NO	−0.4	284.29468	[M + H]^+^	alkaloids	[63]
66	21.770	–	1-stearoylglycerol	C_21_H_42_O_4_	0.76	359.31583	[M + H]^+^	esters	

–: not detected.

**Table 3:** IC_50_ values of the DDPH and ABTS antioxidant activities of methanol extracts of branches and leaves of *Z. myriacanthum* compared with those of ascorbic acid.

Samples	IC_50_ (μg/mL)	
	ABTS	DPPH
Leaves	7.12 ± 0.257 ^※^	1.22 × 10^2^ ± 5.01 ^※^
Branches	5.54 × 10^1^ ± 4.34 ^※^	2.93 × 10^3^ ± 8.43 × 10^1 ※^
Ascorbic acid	6.12 × 10^−3^ ± 1.76 × 10^−3 ∆^	8.12 ± 4.20 × 10^−2 ∆^

^※^: represents the mass of dry material powder contained per 1 mL of solvent; ^∆^: represents the mass of the compound contained in each 1 mL of solvent.

## Data Availability

Not applicable.

## Supplementary Materials

The following supporting information can be downloaded at: https://www.mdpi.com/article/10.3390/molecules28155631/s1, Figures S1 and S2: GC-MS chromatograms of *Zanthoxylum myriacanthum*; Figures S3–S6: UPLC-Q-Orbitrap HRMS chromatograms of *Zanthoxylum myriacanthum*. Figures S7 and S8: The structures of non-volatile and volatile components shared by branches and leaves of *Zanthoxylum myriacanthum*, respectively.

## Sample Availability

Not available.
