# Supplementary material for: Comparative Analysis of Chemical Composition of Zanthoxylum myriacanthum Branches and Leaves by GC-MS and UPLC-Q-Orbitrap HRMS, and Evaluation of Their Antioxidant Activities
Source: Molecules. 2023 Jul 25;28(15):5631. doi: 10.3390/molecules28155631 (PMC10419930; doi:10.3390/molecules28155631)
Supplement: Supplementary file 1 [file molecules-28-05631-s001.zip › molecules-2496035-supplementary.pdf]

Comparative Analysis of Chemical Composition of *Zanthoxylum myriacanthum* Branches and Leaves by GC-MS and UPLC-Q-Orbitrap HRMS, and Evaluation of their Antioxidant Activities

Wei Dai [1,*], Liangqian Zhang [2], Liping Dai [3], Yuan Tian [2], Xinger Ye [3], Sina Wang [1], Jingtao Li [1] and Qi Wang [2,*]

[1] Experimental Center of Yunfu Campus, Guangdong Pharmaceutical University, Yunfu 527325, China; wangsn@gdpu.edu.cn (S.W.); kingtal@gdpu.edu.cn (J.L.)
[2] Shihezi University College of Pharmacy/Key Laboratory of Xinjiang Phytomedicine Resource and Utilization, Ministry of Education, Shihezi, 832003, China; 17799930579@163.com (L.Z.); 15700973063@163.com (Y.T.)
[3] College of Traditional Chinese Medicine Resources, Guangdong Pharmaceutical University, Yunfu 527325, China; 16680128026@163.com (L.D.); xeye@gdpu.edu.cn (X.Y.)
* Correspondence: dai_gdpu_2018@gdpu.edu.cn (W.D.); qiw16@163.com (Q.W.)

Figure S1. GC-MS chromatograms of branches of *Zanthoxylum myriacanthum*
Figure S2. GC-MS chromatograms of leaves of *Zanthoxylum myriacanthum*
Figure S3. UPLC-Q-Orbitrap HRMS chromatograms of branches *Zanthoxylum myriacanthum* in negative ion mode
Figure S4. UPLC-Q-Orbitrap HRMS chromatograms of branches *Zanthoxylum myriacanthum* in positive ion mode
Figure S5. UPLC-Q-Orbitrap HRMS chromatograms of leaves *Zanthoxylum myriacanthum* in negative ion mode
Figure S6. UPLC-Q-Orbitrap HRMS chromatograms of leaves *Zanthoxylum myriacanthum* in positive ion mode
Figure S7. *Zanthoxylum myriacanthum* leaves and branches identified identical volatile structures
Figure S8. *Zanthoxylum myriacanthum* leaves and branches identified identical non–volatile structures

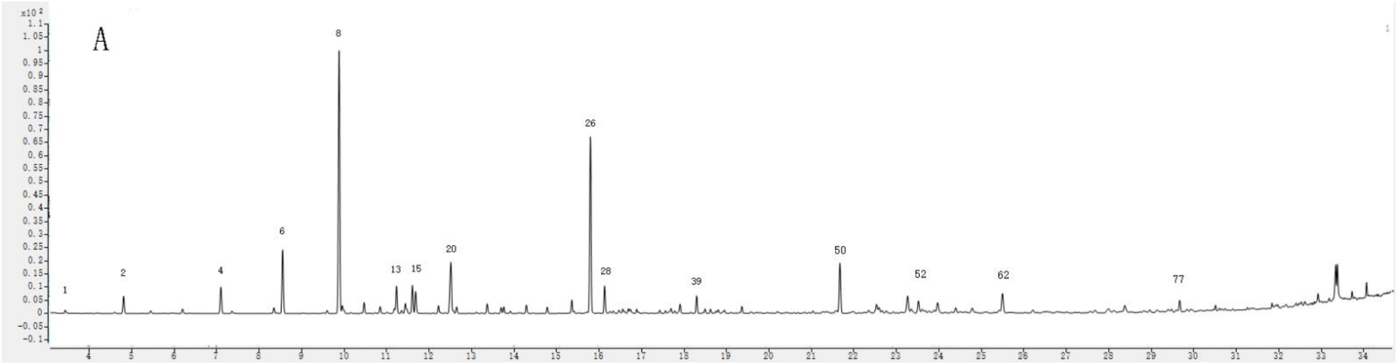

Figure S1. GC-MS chromatograms of branches of *Zanthoxylum myriacanthum*

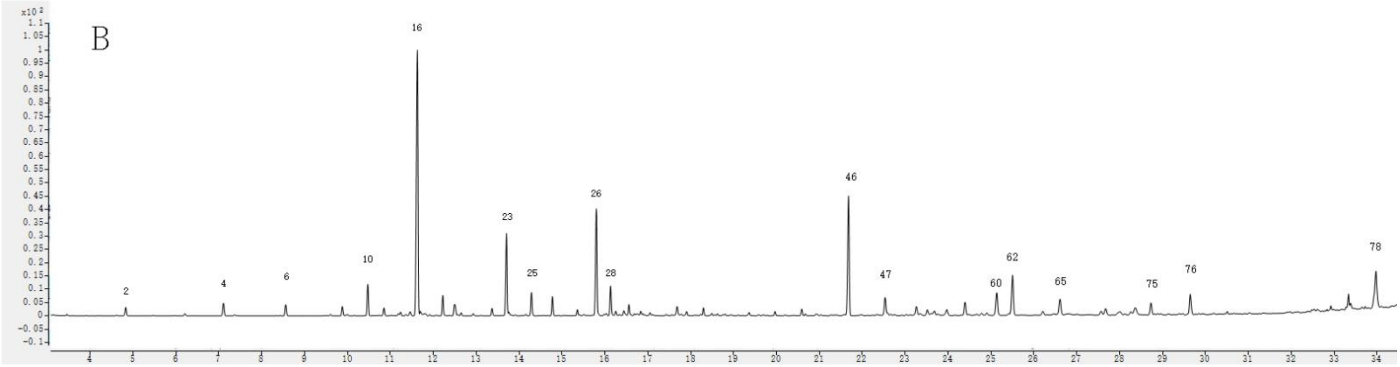

Figure S2. GC-MS chromatograms of leaves of *Zanthoxylum myriacanthum*

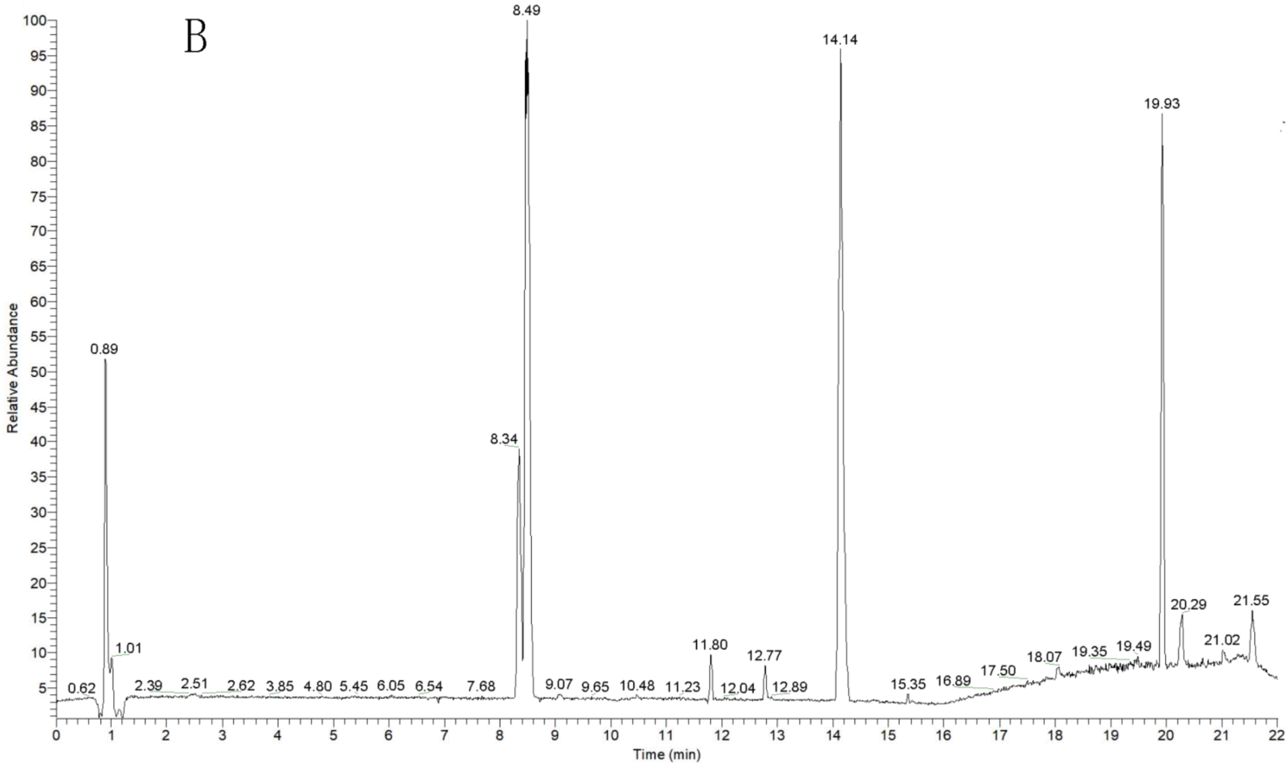

Figure S3. UPLC-Q-Orbitrap HRMS chromatograms of branches *Zanthoxylum myriacanthum* in negative ion mode

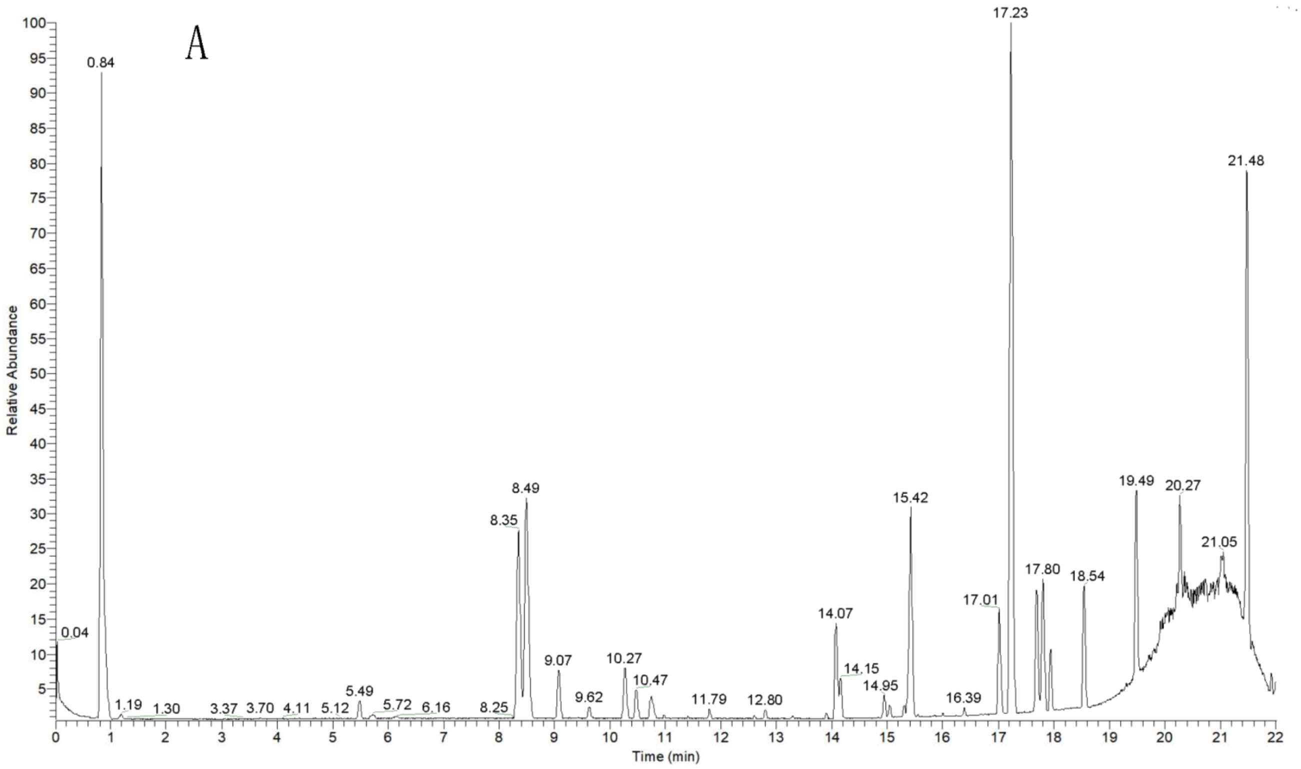

Figure S4. UPLC-Q-Orbitrap HRMS chromatograms of branches *Zanthoxylum myriacanthum* in positive ion mode

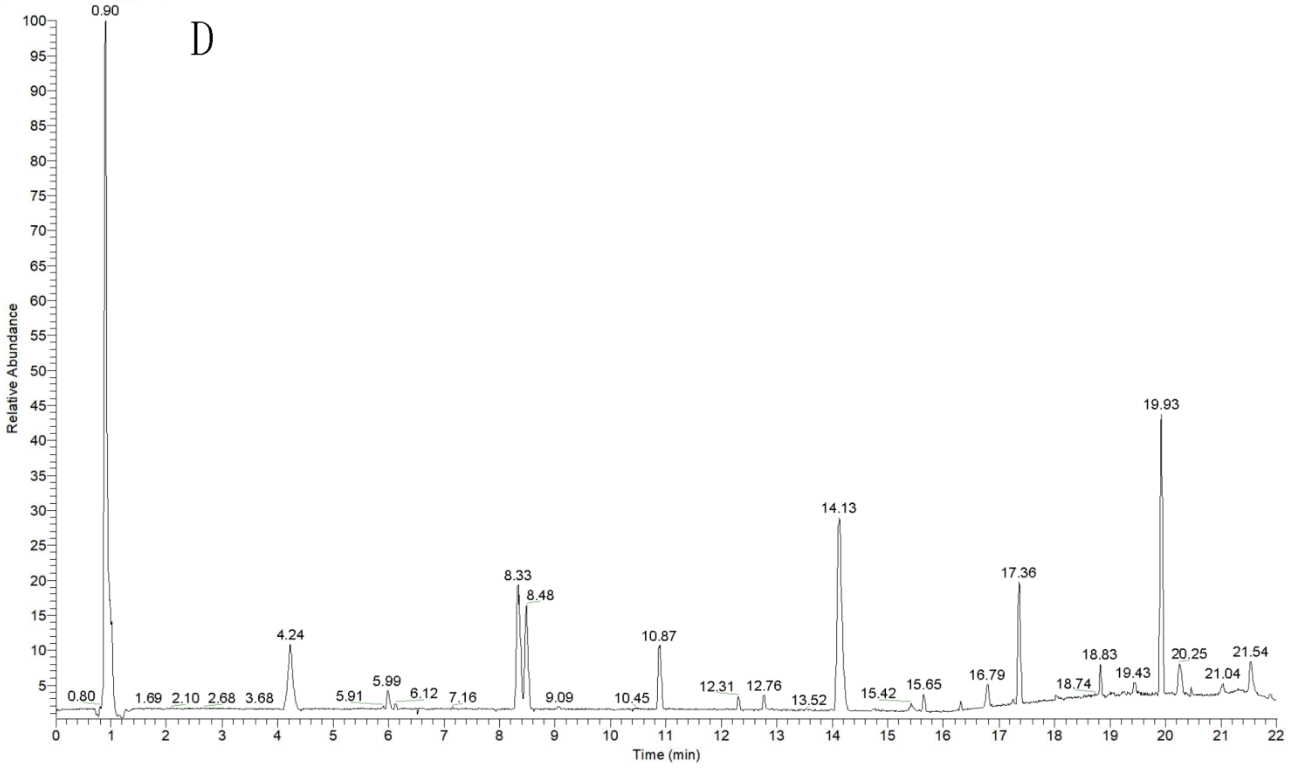

Figure S5. UPLC-Q-Orbitrap HRMS chromatograms of leaves *Zanthoxylum myriacanthum* in negative ion mode

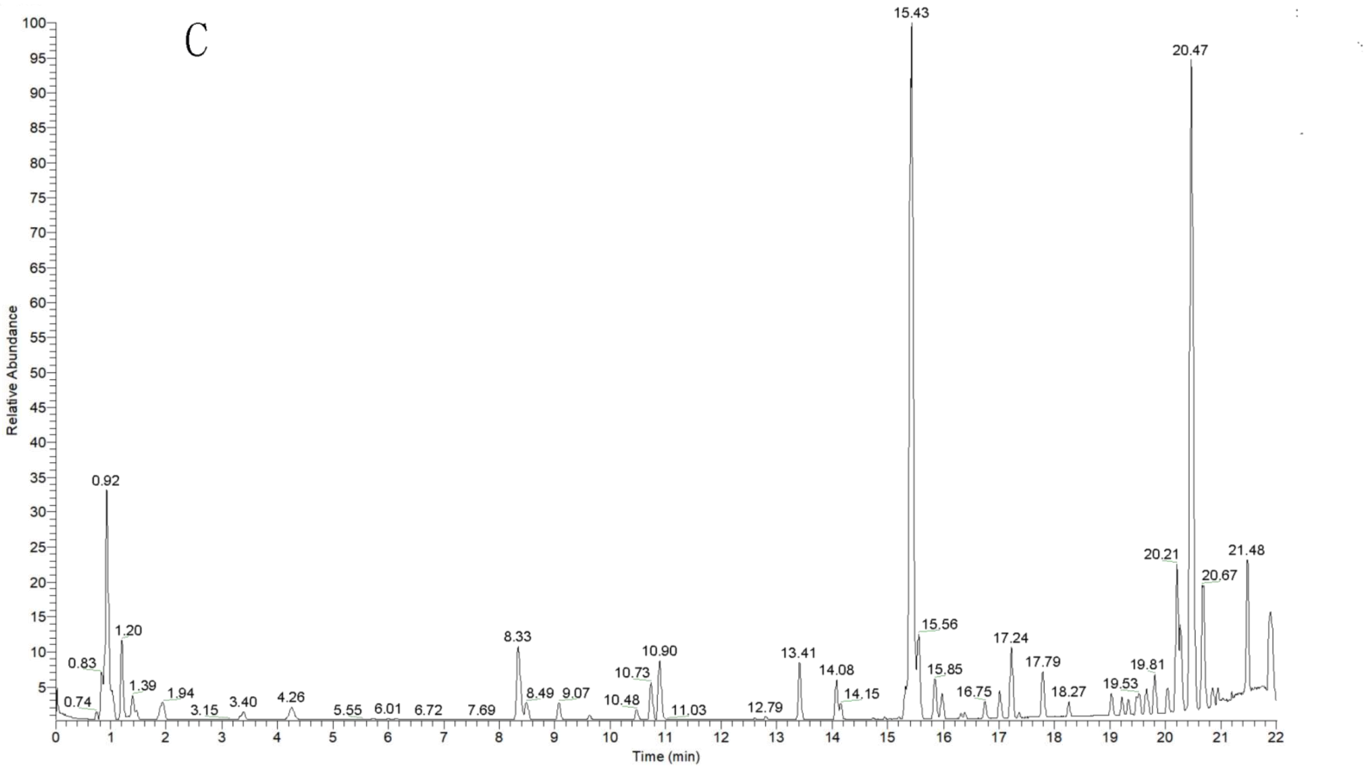

Figure S6. UPLC-Q-Orbitrap HRMS chromatograms of leaves *Zanthoxylum myriacanthum* in positive ion mode

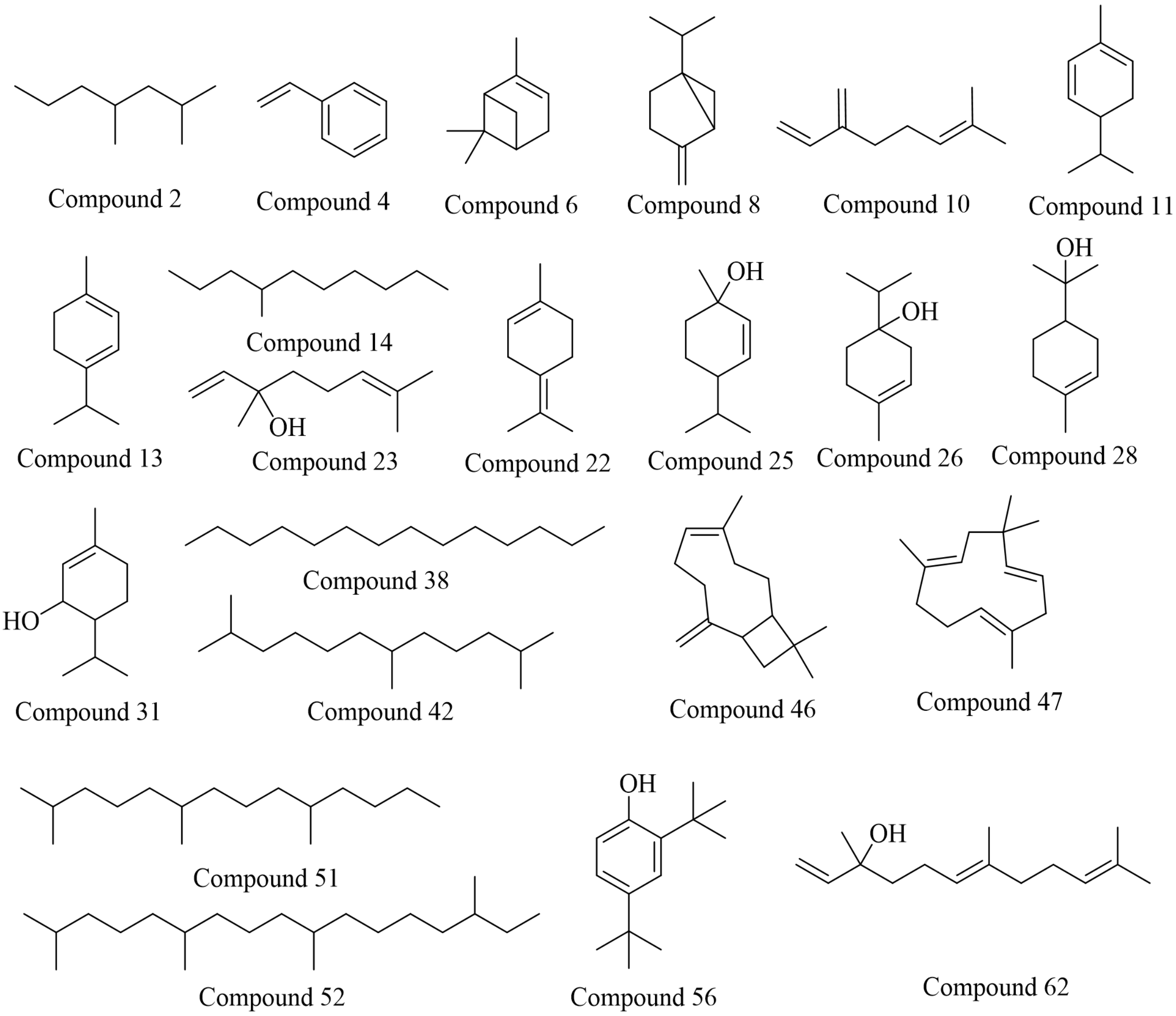

Figure S7. *Zanthoxylum myriacanthum* leaves and branches identified identical volatile structures

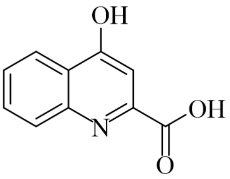
Compound 5

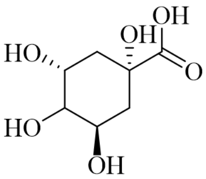
Compound 7

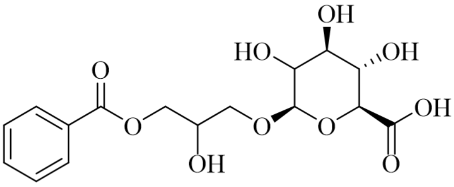
Compound 16

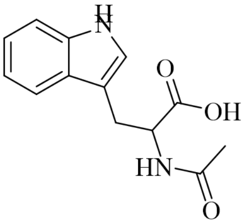
Compound 23

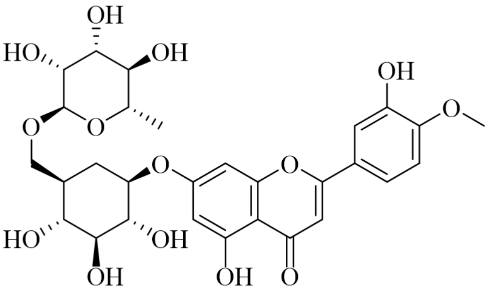
Compound 32

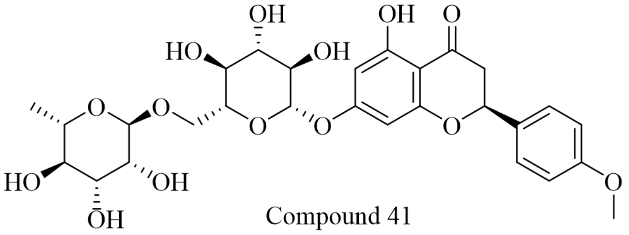
Compound 41

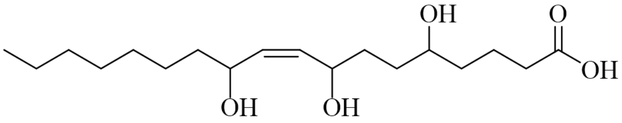
Compound 45

Figure S8. *Zanthoxylum myriacanthum* leaves and branches identified identical non–volatile structures
